# Phonon-mediated repulsion, sharp transitions and (quasi)self-trapping in the extended Peierls-Hubbard model

**DOI:** 10.1038/s41598-017-01228-y

**Published:** 2017-04-26

**Authors:** J. Sous, M. Chakraborty, C. P. J. Adolphs, R. V. Krems, M. Berciu

**Affiliations:** 1 Department of Physics and Astronomy, University of British Columbia, Vancouver, British, Columbia V6T 1Z1 Canada; 2 Stewart Blusson Quantum Matter Institute, University of British Columbia, Vancouver, British, Columbia V6T 1Z4 Canada; 3 Department of Physics, Indian Institute of Technology, Kharagpur, India; 4 Department of Chemistry, University of British Columbia, Vancouver, British, Columbia V6T 1Z1 Canada

## Abstract

We study two identical fermions, or two hard-core bosons, in an infinite chain and coupled to phonons by interactions that modulate their hopping as described by the Peierls/Su-Schrieffer-Heeger (SSH) model. We show that exchange of phonons generates effective nearest-neighbor *repulsion* between particles and also gives rise to interactions that move the pair as a whole. The two-polaron phase diagram exhibits two sharp transitions, leading to light dimers at strong coupling and the flattening of the dimer dispersion at some critical values of the parameters. This dimer (quasi)self-trapping occurs at coupling strengths where single polarons are mobile. This illustrates that, depending on the strength of the phonon-mediated interactions, the coupling to phonons may completely suppress or strongly enhance quantum transport of correlated particles.

## Introduction

Strongly correlated quantum materials exhibit rich physics with many features yet to be understood. Correlated lattice systems are modeled by the extended Hubbard model, which includes inter-site interactions giving rise to interesting physics such as superfluid - Mott insulator transitions^1^, antiferromagnetism^2, 3^, high-Tc superconductivity^4^, twisted superfluidity^5^, supersolids^6^. However, the extended Hubbard model does not include interactions with phonons, which are essential for quantum materials. Here, we show that the interplay of the extended Hubbard interactions with phonon-mediated couplings leads to new unique features, such as self-trapping of correlated pairs and the formation of light (mobile) dimers in the regime of strong interactions, both between the particles and with phonons.

A particle (electron, exciton, etc.) dressed with phonons is a polaron. If phonons modulate the on-site energy of the particle, as is the case for electrons in ionic lattices, polarons can be viewed as the bare particle dragging a cloud of phonons. Such polarons are always heavier than the bare particle^7–15^. On the other hand, phonons also modulate the hopping of the particle between sites. Such interactions are important for electrons in conjugated polyenes, where they are described by the Su-Schrieffer-Heeger (SSH) model^16–20^, or for excitons in molecular solids, where they are described by the Peierls model. Polarons arising from the SSH/Peierls interactions exhibit sharp transitions^21^ into strong-coupling regimes where the polaron (dressed particle) is *lighter* than the bare particle^21–26^.

The interplay of the SSH/Peierls couplings and the extended Hubbard interactions may alter the behaviour of strongly correlated quantum systems. For example, in the limit of half-filling, an interplay of phonon-mediated attraction with repulsive Hubbard interactions is known to lead to a competition between the Mott-insulator and Peierls-insulator phases^27^. Here, we consider polarons arising in the two-particle limit of an extended Hubbard model coupled to phonons through the SSH/Peierls couplings. This is critical for understanding quantum transport of interacting excitons in devices based on organic semiconductors (such as low-temperature solar cells)^28, 29^ and the prospects of observing the Mott-insulator/Peierls-insulator competition with highly controllable ultracold atoms/molecules systems, which require understanding of emergent interactions in the few-particle limit. The extended Peierls-Hubbard model can be realized for hard-core bosons with ions in rf-traps^30–33^, Rydberg atoms exchanging excitations^34–38^, self-assembled ultracold dipolar crystals^39–41^, arrays of polar molecules trapped in optical lattices^42, 43^, arrays of superconducting qubits^44–49^, and J-aggregates^50^. Similar physics may also arise in the context of interacting impurities in a Fermi degenerate gas^51–53^ or Bose-Einstein condensates^54–59^ of ultracold atoms. Motivated by these experiments, we consider identical fermions/hard-core bosons and show that the interplay between particle statistics, particle interactions and coupling to phonons leads to unique features such as *phonon-mediated repulsion* and *sharp transitions* in the ground-state properties of dimers including one suggestive of *self-trapping*.

### Model

We consider two identical fermions (fermionic atoms in the same internal state), or equivalently, two hard-core bosons, placed in an infinite chain^60, 61^ described by the Hamiltonian ${\mathcal H} ={ {\mathcal H} }_{{\rm{p}}}+{ {\mathcal H} }_{{\rm{ph}}}+\hat{V}$, where:

1 $${H}_{{\rm{p}}}=-t\sum _{i}({c}_{i}^{\dagger }{c}_{i+1}+h\mathrm{.}c\mathrm{.})+U\sum _{i}{\hat{n}}_{i}{\hat{n}}_{i+1}$$

is the extended Hubbard model of the bare particles with infinite on-site repulsion, ${ {\mathcal H} }_{{\rm{ph}}}={\rm{\Omega }}{\sum }_{i}{b}_{i}^{\dagger }{b}_{i}$ is the phonon Hamiltonian (in units of $\hslash =1$), and

2 $$\hat{V}=g\sum _{i}({c}_{i}^{\dagger }{c}_{i+1}+h\mathrm{.}c\mathrm{.})({b}_{i}^{\dagger }+{b}_{i}-{b}_{i+1}^{\dagger }-{b}_{i+1})$$

is the Peierls/SSH particle - phonon coupling^21^. Here, *i* is the site index, ${\hat{n}}_{i}={c}_{i}^{\dagger }{c}_{i}$, *U* is the strength of the bare nearest-neighbor (NN) interactions and Ω is the phonon frequency. We characterize the particle - phonon effective coupling by the dimensionless parameter *λ* = 2*g* ^2^/(Ω*t*).

## Methods

We use two methods to investigate this problem. The first is variational exact diagonalization (VED), a well-established, unbiased numerical method, where the variational basis set is expanded systematically, starting from the Bloch state for two adjacent particles and zero phonons^62–64^. The second method is based on the Momentum Average (MA) approximation, a quasi-analytical variational method that has been shown to be accurate for polarons^65–67^, including SSH polarons^21^. Here, we generalize MA to study bound dimers by allowing a variational space where the two particles are either in adjacent sites or two sites apart, interacting with a phonon cloud spread over at most three adjacent sites (for more details, see the Supplementary Information); we comment more on these choices below.

## Results

First, we set *U* = 0 and study whether exchange of phonons suffices to bind two SSH polarons into a bipolaron. For reference, we note that equivalent 1D models with long(er)-range on-site energy-modulating couplings, such as the screened and unscreened Fröhlich couplings, show the appearance of stable bipolarons; for on-site Holstein coupling, such bipolarons do not form refs 63, 64 and 68.

We find that for *U* = 0, bipolarons do not form for any coupling *λ*. To understand the implications of this result, note that the bare particles (*λ* = 0) bind only for *U* ≤ −2*t*. This attraction is needed to compensate for the loss of kinetic energy^69^. The SSH polaron dispersion – and hence its kinetic energy – remains significant at all particle - phonon couplings. Our results thus show that the phonon-mediated interaction is insufficient to compensate for the kinetic energy that would be lost upon binding.

**Figure 1:**
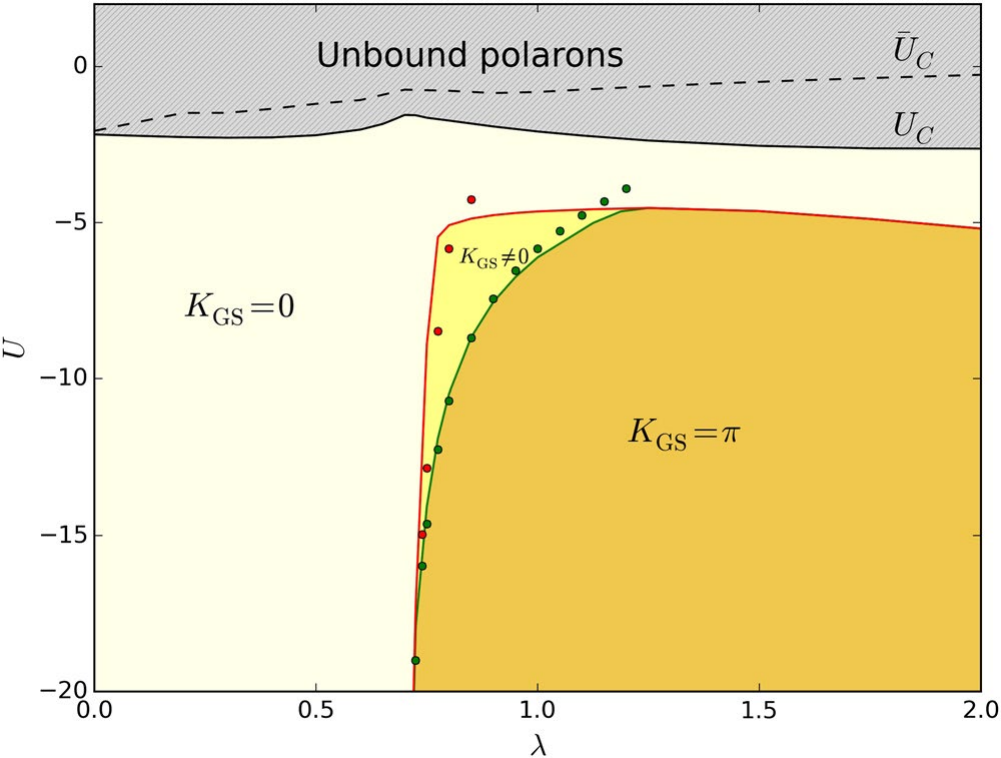
Two-polaron phase diagram at *t* = 1, Ω = 3. The solid black line shows *U*_*C*_(*λ*) below which stable bound states form, while the dashed line shows ${\bar{U}}_{C}(\lambda )$; the difference between the two is the strength of the phonon-mediated interaction. Note that ${U}_{C} < {\bar{U}}_{C}$, which means that this interaction is repulsive. The red and green lines mark the sharp transitions of the bound dimer’s GS. The lines are the VED results and the corresponding symbols are the MA results.

To characterize quantitatively this phonon-mediated interaction, we compute the values of *U* = *U*_*C*_(*λ*) corresponding to the onset of stable bound state (we define the bound dimer to be stable if its ground state energy is below the two-polaron continuum). We then compare *U*_*C*_(*λ*) with ${\bar{U}}_{C}(\lambda )$, defined as the NN attraction needed to bind two hard-core particles with dispersions identical to those of single SSH polarons. This latter model mimics the renormalization of the dispersion due to each particle creating and interacting with its own cloud of phonons, but excludes the effective interactions due to phonon exchange between the clouds. The phonon exchange occurs in the full model, so $|{\bar{U}}_{C}(\lambda )|-|{U}_{C}(\lambda )|$ is an estimate of the phonon-mediated NN attraction between polarons. Figure 1 shows that $|{U}_{C}(\lambda )| > |{\bar{U}}_{C}(\lambda )|$ for all *λ*. This means that the phonon-mediated interaction is in fact *strongly repulsive*, in stark contrast to what is observed for conventional polaron models^63, 64, 68^.

This surprising result can be explained by considering the limit ${\rm{\Omega }}\gg |t|,|g|$ within perturbation theory (details in Supplementary Information). Projecting out high-energy states with one or more phonons, the effective Hamiltonian for a single polaron becomes

3 $${\hat{h}}_{1}=\sum _{i}(-t{c}_{i}^{\dagger }{c}_{i+1}+{t}_{2}{c}_{i}^{\dagger }{c}_{i+2}+h\mathrm{.}c\mathrm{.})-{\varepsilon }_{0}\sum _{i}{\hat{n}}_{i}\mathrm{.}$$

The small cloud that forms in this limit does not renormalize the NN hopping but it mediates a next-nearest-neighbor (NNN) hopping *t*_2_ = *g* ^2^/Ω = *λt*/2 through the process ${c}_{i}^{\dagger }|0\rangle \mathop{\Rightarrow }\limits^{\hat{V}}{c}_{i+1}^{\dagger }{b}_{i+1}^{\dagger }|0\rangle \mathop{\Rightarrow }\limits^{\hat{V}}{c}_{i+2}^{\dagger }|0\rangle$. The four processes ${c}_{i}^{\dagger }|0\rangle \mathop{\Rightarrow }\limits^{\hat{V}}{c}_{i\pm 1}^{\dagger }{b}_{i\pm 1}^{\dagger }|0\rangle \mathop{\Rightarrow }\limits^{\hat{V}}{c}_{i}^{\dagger }|0\rangle$ and ${c}_{i}^{\dagger }|0\rangle \mathop{\Rightarrow }\limits^{\hat{V}}{c}_{i\pm 1}^{\dagger }{b}_{i}^{\dagger }|0\rangle \mathop{\Rightarrow }\limits^{\hat{V}}{c}_{i}^{\dagger }|0\rangle$ explain the polaron formation energy ${\epsilon }_{0}=4{g}^{2}/{\rm{\Omega }}$ ^21^. The resulting polaron dispersion ${E}_{P}(k)=-{\epsilon }_{0}-2t\,\cos (k)+2{t}_{2}\,\cos (2k)$ is dominated by the NNN hoping at large *λ*; this explains both the transition, at *λ* = 1/2, of the polaron ground state (GS) momentum from *k* = 0 to a finite value that smoothly goes to *k* = *π*/2, and why the polaron remains light at large *λ* (for more discussion, see ref. 21). Repeating the calculation for two particles, we find the corresponding effective Hamiltonian to be

4 $${\hat{h}}_{2}={\hat{h}}_{1}+{\epsilon }_{0}\sum _{i}{\hat{n}}_{i}{\hat{n}}_{i+1},$$

illustrating the appearance of phonon-mediated NN repulsion. Its origin can be explained as follows: if the polarons are *δ* ≥ 2 sites apart, each lowers its energy by ${\epsilon }_{0}$ through hops to its adjacent sites and back, accompanied by virtual phonon emission and absorption, as explained above. However, if the polarons are on adjacent sites, then Fermi statistics blocks half of these processes, *i.e*. each particle can only lower its energy by ${\epsilon }_{0}/2$. The energy cost for polarons to be adjacent is, thus, ${\epsilon }_{0}=2\lambda t$.

It is very important to note that ${\hat{h}}_{2}$ also includes terms such as ${c}_{i}^{\dagger }{c}_{i+1}^{\dagger }|0\rangle \mathop{\Rightarrow }\limits^{{\hat{h}}_{2}}{c}_{i+1}^{\dagger }{c}_{i+2}^{\dagger }|0\rangle$. However, NNN hopping of one particle past the other is forbidden by statistics (the particle at *i* cannot emit a phonon and move to *i* + 1 because that site is occupied). Instead, these terms describe both particles moving through ${c}_{i}^{\dagger }{c}_{i+1}^{\dagger }|0\rangle \mathop{\Rightarrow }\limits^{\hat{V}}{c}_{i}^{\dagger }{b}_{i+1}^{\dagger }{c}_{i+2}^{\dagger }|0\rangle \mathop{\Rightarrow }\limits^{\hat{V}}{c}_{i+1}^{\dagger }{c}_{i+2}^{\dagger }|0\rangle$. In other words, instead of one particle hopping over the other, which is forbidden, each particle moves by one site and a phonon is exchanged in the process. Thus, this term is also a phonon-mediated effective interaction which would be absent if phonons could not be exchanged between particles. In the large Ω limit it happens to precisely compensate for the NNN hopping forbidden by the particles’ statistics, but that is not likely to be the case throughout the parameter space. This shows that the functional form of the effective phonon-mediated interaction must also contain such “pair-hopping” terms in addition to the NN repulsion. Such terms do not appear in models where phonons modulate the on-site particle energy (e.g., Holstein and Fröhlich models).

**Figure 2:**
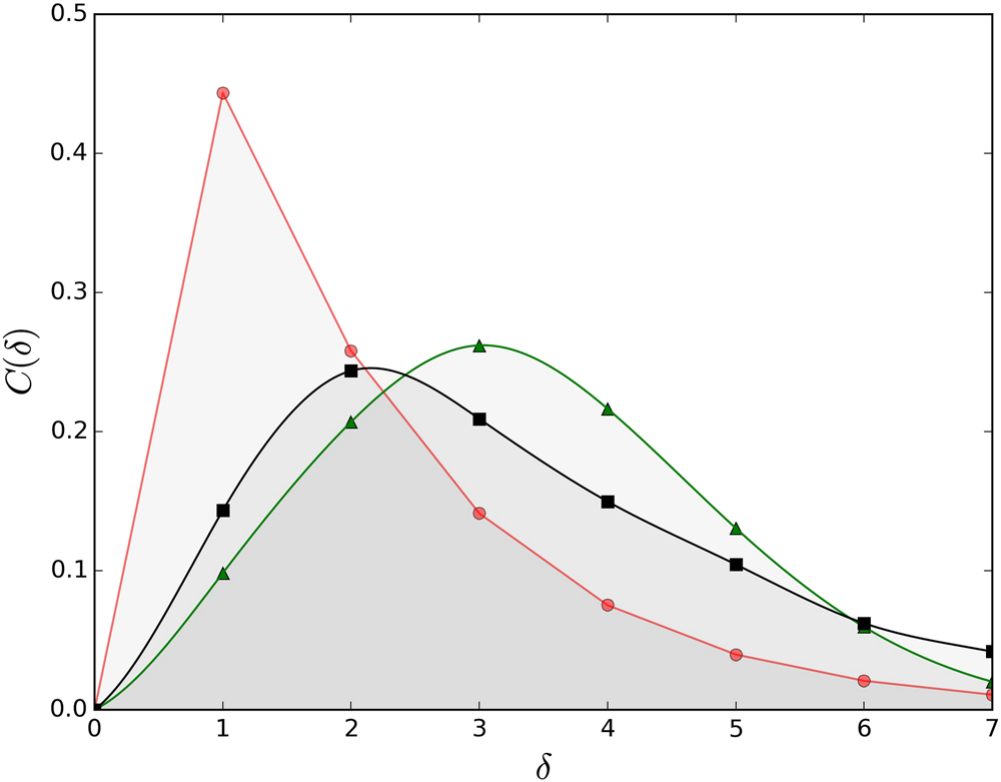
Correlation between the two particles $C(\delta )=\langle {{\rm{\Psi }}}_{GS}|1/N{\sum }_{i}{\hat{n}}_{i}{\hat{n}}_{i+\delta }|{{\rm{\Psi }}}_{GS}\rangle$ *vs* separation *δ* for *U* = *U*_*C*_(*λ*) − 0.5, *t* = 1, Ω = 3 and *λ* = 0.1 (red circles), *λ* = 0.7 (green triangles), *λ* = 2.0 (black squares).

For smaller values of Ω, the phonon clouds have more phonons and are more extended spatially, and thus can mediate longer-range effective interactions and hopping. Indeed, as shown in Fig. 2 for Ω = 3 and *U* = *U*_*C*_(*λ*) − 0.5, *i.e*. just inside the dimer stability region, the bound particles favor adjacent locations only for *λ* → 0. At moderate and strong couplings they are found with highest probability to be 2 or even 3 sites apart, even though the bare attraction is NN only. This suggests that the strong phonon-mediated NN repulsion is supplemented by longer range effective attraction, and/or that binding is due to kinetic energy gained through phonon-mediated “pair-hopping” terms such as the one discussed above.

**Figure 3:**
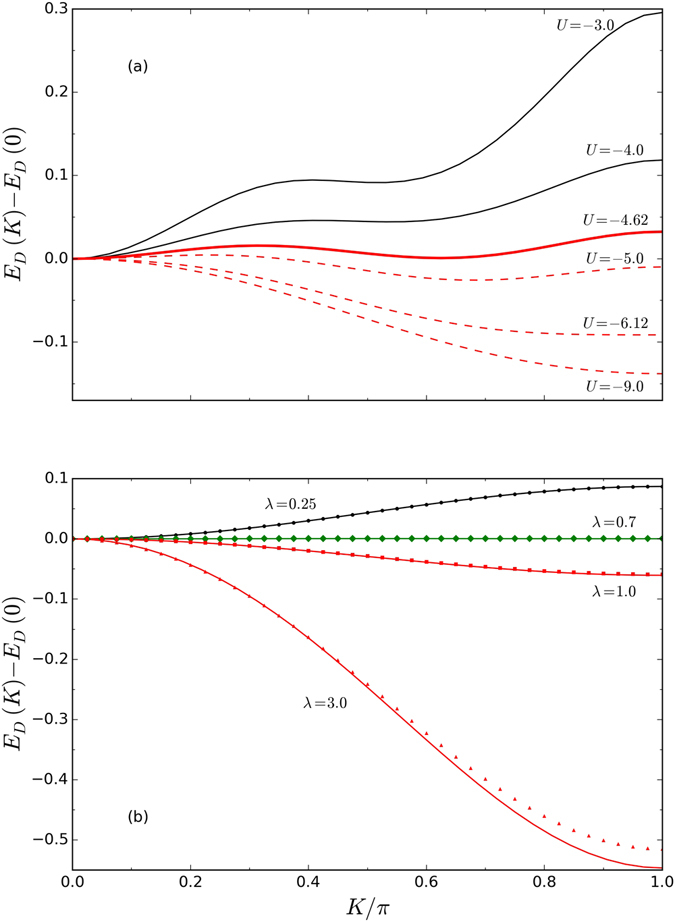
Dimer dispersion *E*_*D*_(*K*) − *E*_*D*_(0) for (**a**) *λ* = 1 and various values of *U*; and (**b**) *U* = −30 and various values of *λ*. In both cases *t* = 1, Ω = 3. The lines are the VED results and the symbols are the MA results. Note the sharp transitions of the GS momentum from *K*_GS_ = 0 to *K*_GS_ > 0 in both cases.

We now examine the properties of dimers formed when *U* is sufficiently large to balance the phonon-mediated repulsion and the loss of kinetic energy. Figure 3 shows the dimer dispersion, *E*_*D*_(*K*) as a function of *U* and *λ*, illustrating two unique features of dimers arising from the SSH coupling. At low |*U*| and/or *λ*, the dimer ground state has momentum *K*_*GS*_ = 0. As *λ* and/or |*U*| increases, there is a sharp transition to a GS momentum *K* > 0. Figure 3(a) shows that with increasing |*U*|, the dimer dispersion develops a rather unusual shape with a second local minimum appearing at a finite momentum. At *U* ≈ −4.62 *t* this minimum becomes degenerate with that at *K* = 0, and the dimer ground-state momentum jumps discontinuously to *K*_*GS*_ ≈ 0.6*π* and then continues to increase with increasing |*U*|. There is a second sharp transition to *K*_*GS*_ = *π* at *U* = −6.12 *t*. These curves are at a fixed *λ* so the polaron dispersion is unchanged. The change in the dimer dispersion (and in *K*_*GS*_) is therefore due to forcing the bound polarons closer, as |*U*| increases.

In Fig. 3(b) we follow the evolution of *E*_*D*_(*K*) with *λ* for a fixed *U* = −30 *t*. At small *λ* we see a rather heavy dimer with *K*_*GS*_ = 0, as expected because in this limit the non-interacting polarons are quite heavy and with *E*_*P*_(*k*) increasing monotonically with *k* ^21^. With increasing *λ* the effective dimer mass increases fast and the dispersion becomes flat. At a value *λ* ^*^ ≈ 0.7 the minimum jumps discontinuously to *K*_*GS*_ = *π*. It stays there with further increase in *λ*, but the bandwidth increases dramatically as the phonon-mediated pair-hopping terms become dominant, thus making the dimers light at strong coupling.

Figure 1 illustrates the locations of these sharp transitions for the dimers in the extended Peierls-Hubbard model on the *U*-*λ* phase diagram. To the best of our knowledge, this is the first observation of such sharp transitions of the two–polaron ground state. They never occur in Holstein or Fröhlich models, where the bipolarons always have *K*_*GS*_ = 0^63, 64, 68^.

The second unique feature illustrated in Fig. 3 is the flattening of the dimer dispersion at *λ* = *λ* ^*^ ≈ 0.7, suggestive of *self-trapping*: here the dimers are essentially localized even though the single polarons have finite bandwidth. This behavior can be understood qualitatively as follows. For small *λ*, the polaron dispersion is dominated by its NN hopping. A large |*U*| can bind the polarons only when they are on adjacent sites. When acting on such a configuration, NN hopping moves the particles two sites apart to an energetically expensive configuration. As a result, the effective dimer dispersion acquires a term $\sim-{t}^{2}/|U|\cos (K)$, which favors *K*_*GS*_ = 0 (see Supplementary Information). On the other hand, at finite *λ*, the “pair-hopping” process moving the NN pair as a whole also becomes active and contributes a term of order 2*t*_2_cos(*K*) to the dimer dispersion (see Supplementary Information); this term favors *K*_*GS*_ = *π*. At *λ* = *λ* ^*^ the two terms cancel and the bandwidth collapses. However, numerical simulations cannot guarantee that the bandwidth is precisely zero, and we do not have theoretical arguments why the longer range hopping should also vanish at *λ* ^*^. This is why we prefer to use the more conservative term of (quasi) self-trapping.

Before concluding, we highlight another accomplishment of this work, demonstrated by Figs 1 and 3, namely the successful generalization of the MA approximation to bipolaron-type problems. The variational space we implemented here (see Supplementary Information) is designed, by construction, to describe strongly bound polarons. Indeed, the MA predictions are in quantitative agreement with VED in this limit. The MA results are also qualitatively correct near *U*_*C*_(*λ*) (not shown), but its accuracy is much poorer for weakly bound polarons. A suitable increase of the variational space is necessary to improve the accuracy of the MA approximation for weakly bound states. This can be done in a rather straightforward way and promises to establish MA as an equally valuable and efficient method for the study of bipolarons as it is for polarons.

## Discussion

To summarize, we showed that dressing interacting particles by phonons through SSH/Peierls couplings leads to very rich two-polaron physics, qualitatively different from what is known for conventional polaron models. We showed that for bare particles with the statistics of identical fermions or of hard-core bosons, the phonon-mediated interactions are repulsive, contradicting the conventional view that phonons act as “glue” for quasiparticles. We showed that the “pair-hopping” terms, which are also mediated by phonon-exchange and can only arise in models with phonons modulating the particle hopping, play a major role, leading to *sharp transitions* of the bound dimer’s ground state. We also observe the collapse of the dimer’s dispersion at phonon coupling strength *λ* ^*^ where the single polarons are mobile, suggestive of a self-trapping transition.

As discussed in the previous section, all these new observations rest on the interplay of two generic features: hard-core statistics of bare particles and off-diagonal, hopping-dependent particle-phonon couplings. As such, these results apply to a wide range of systems and have far-reaching implications for complex quantum systems of interacting dressed particles. The Hamiltonians considered here describe the interactions of small excitons coupled to phonons, particularly relevant to molecular crystals and organics semiconductors^28, 29^. Moreover, the hopping-dependent interactions with phonons, such as the one described by Eq. (2), are generally present in all materials. They may not always be dominant but, because they lead to qualitatively distinct behaviour of the resulting dressed particles, our work raises an important question of how the interplay of the coupling terms in Eq. (2) with conventional phonon-induced interactions changes the dynamics of polarons. As we showed in previous work^43^, a perturbative admixture of the hopping-dependent interactions may lead to non-perturbative changes of the single polaron dispersion. If a similar effect happens for dimers or bipolarons at experimentally relevant interaction parameters, many of the long-standing questions in polaron physics must be re-visited to account for the hopping-dependent interactions with phonons.

In addition, our results suggest that soft-core bosons and/or singlet fermions may form highly mobile bipolarons with sharp transitions even in the limit of vanishing *U*; we are currently investigating this. Also, many of these features are expected to apply to systems with more particles. The “pair-hopping” terms must be equally important for few-polaron ensembles, suggesting that the ground state of few-polaron states must also exhibit sharp transitions and, perhaps, localization (self-trapping).

## Electronic supplementary material

Supplementary Material for “Phonon-mediated repulsion, sharp transitions and (quasi)self-trapping in the extended Peierls-Hubbard model”
