# Supplementary material for: Phonon-mediated repulsion, sharp transitions and (quasi)self-trapping in the extended Peierls-Hubbard model
Source: Sci Rep. 2017 Apr 26;7:1169. doi: 10.1038/s41598-017-01228-y (PMC5430795; doi:10.1038/s41598-017-01228-y)
Supplement: Supplementary file 1 — Supplementary Material for “Phonon-mediated repulsion, sharp transitions and (quasi)self-trapping in the extended Peierls-Hubbard model” [file 41598_2017_1228_MOESM1_ESM.pdf]

Supplementary Material for
"Phonon-mediated repulsion, sharp transitions and (quasi)self-trapping in the extended Peierls-Hubbard model"

J. Sous, M. Chakraborty, C. P. J. Adolphs, R. V. Krems, and M. Berciu

(Dated: February 26, 2017)

MOMENTUM AVERAGE (MA) APPROXIMATION - TECHNICAL DETAILS

The Momentum Average (MA) [1–3] approximation is a non-perturbative quasi-analytical technique designed to solve the equation of motion for the relevant Green's function $G(k,\omega) = \langle k|(\omega - \mathcal{H} + i\eta)^{-1}|k\rangle$ in the Bogoliubov-Born-Green-Kirkwood-Yvon (BBKGY) hierarchy. The hierarchy consists of an infinite set of coupled equations which are impossible to solve exactly. By neglecting exponentially small contributions in the expansion, one simplifies the equations of motion to a form that is readily solvable numerically. The guide to approximating the hierarchy follows from the variational meaning of MA: essentially one solves the problem in a variational subspace.

The choice of the variational space depends on the details of the Hamiltonian and state(s) of interest [2]. For the Holstein model, a one-site phonon cloud suffices to provides accurate results for single polarons [1, 2] and for S0 bipolarons [5]. S0 bipolarons are single-site strongly bound bipolarons. Taken together with the local nature of the Holstein coupling, this explains why a one-site phonon cloud is accurate to describe such states. For the Edwards and SSH models, the coupling to phonons is non-local and therefore a bigger cloud is required to yield accurate results. A three-site phonon cloud MA has been shown to be very accurate for such models [4, 6].

In this work, we generalize MA to study strongly bound two-particle states in the extended Peierls/Su-Schrieffer-Heeger (SSH)–Hubbard model. We derive the MA equations for two hardcore particles in a three-site phonon cloud and allow the particles to be arbitrarily far from the cloud but at most two sites apart from each other, if a cloud is present. Terms corresponding to the particles being further than two sites apart are expected to contribute significantly only to higher-energy states, if a two-particle state is strongly bound, which is the case of primary interest to us in this work. For weakly bound dimers, the variational space must be extended to include configurations where the particles are further apart. The variational space can be increased systematically until convergence is achieved.

To highlight the method we derive a few representative equations of motion used in the MA formalism developed here [7]. This is achieved using Dyson's identity $\hat{G}(\omega) = \hat{G}_0(\omega) + \hat{G}(\omega)\hat{V}\hat{G}_0(\omega)$ where $\hat{G}(\omega) = (\omega - \mathcal{H} + i\eta)^{-1}$, $\hat{G}_0(\omega) = (\omega - \mathcal{H}_0 + i\eta)^{-1}$ with $\mathcal{H}_0 = \mathcal{H}_p + \mathcal{H}_{ph}$, and $\hat{V}$ is the bare particle - phonon coupling term.

Consider the two-particle propagator $G(K,1,n,\omega) = \langle K,1|\hat{G}(w)|K,n\rangle$ defined for two-

particle states $|K,n\rangle = \sum_i \frac{e^{iK(R_i+na/2)}}{\sqrt{N}} c_i^\dagger c_{i+n}^\dagger |0\rangle$ with the two particles $n \geq 1$ sites apart, and a is the lattice constant. Using Dyson's identity and inserting a resolution of the identity, its exact equation of motion can be written as:

$$
\begin{aligned}
G(K,1,n,\omega) &= G_0(K,1,n,\omega) \\
&\quad + \sum_\eta G_0(K,\eta,n,\omega)\langle K,1|\hat{G}(\omega)\hat{V}|K,\eta\rangle.
\end{aligned}
$$

Note that $G_0(K,1,n,\omega)$ can be calculated exactly analytically in one dimension [8]. Consider now $\hat{V}|K,\eta\rangle$. It consists of states with one phonon plus the particles $\eta \pm 1$ sites apart. Thus, the right-hand side of the exact equation of motion contains an infinite number of terms. Because within MA we restrict the particles to be within two sites of each other when phonons are present, this simplifies the equation of motion to:

$$
\begin{aligned}
G(K,1,n,\omega) &= G_0(K,1,n,\omega) \\
&\quad - ge^{-iKa}G_0(K,2,n,\omega)F_1(-2,1) + gG_0(K,2,n,\omega)F_1(-1,1) \\
&\quad - gG_0(K,2,n,\omega)F_1(0,1) + ge^{iKa}G_0(K,2,n,\omega)F_1(1,1) \\
&\quad - ge^{-3iKa/2}G_0(K,3,n,\omega)F_1(-3,2) \\
&\quad - g[e^{-3iKa/2}G_0(K,1,n,\omega) - e^{-iKa/2}G_0(K,3,n,\omega)]F_1(-2,2) \\
&\quad - 2igsin(Ka/2)G_0(K,1,n,\omega)F_1(-1,2) \\
&\quad + g[e^{3iKa/2}G_0(K,1,n,\omega) - e^{iKa/2}G_0(K,3,n,\omega)]F_1(0,2) \\
&\quad + ge^{3iKa/2}G_0(K,3,n,\omega)F_1(1,2),
\end{aligned} \tag{1}
$$

where $F_1(m,n)$ is shorthand for $F_1(K,m,n,\omega)$ defined as

$$
F_l(K,m,n,\omega) \equiv \sum_i \frac{e^{iKR_i}}{\sqrt{N}} \langle K,1|\hat{G}(\omega)c_{i+m}^\dagger c_{i+m+n}^\dagger b_i^{\dagger l}|0\rangle, \tag{2}
$$

i.e. a generalized one-site cloud propagator. By introducing other appropriate generalized propagators:

$$
F_{l_1,l_2}(K,m,n,\omega) \equiv \sum_i \frac{e^{iKR_i}}{\sqrt{N}} \langle K,1|\hat{G}(\omega)c_{i+m}^\dagger c_{i+m+n}^\dagger b_i^{\dagger l_1} b_{i+1}^{\dagger l_2}|0\rangle, \tag{3}
$$

$$
F_{l_1,l_2,l_3}(K,m,n,\omega) \equiv \sum_i \frac{e^{iKR_i}}{\sqrt{N}} \langle K,1|\hat{G}(\omega)c_{i+m}^\dagger c_{i+m+n}^\dagger b_{i-1}^{\dagger l_1} b_i^{\dagger l_2} b_{i+1}^{\dagger l_3}|0\rangle, \tag{4}
$$

for two-site cloud and three-site cloud configurations respectively, and repeatedly applying the Dyson's identity, one derives the MA equations of motion for the the propagators in Eqs.

(1)-(4). This linear system of coupled equations is solved numerically and the propagator of interest $G(K,1,n,\omega)$ is computed. Dimer bound state properties such as $E_D(K)$ can be extracted from the propagator.

By construction the MA approach developed here is designed to describe strongly bound states accurately. Indeed, the comparison with Variational Exact Diagonalization (VED) results shows that the MA approximation is accurate in this regime. By systematically extending the variational subspace we expect to be able to study weakly-bound dimers as well.

PERTURBATION THEORY (PT) RESULTS IN THE LIMIT $\Omega \gg |t|, |g|$

Single-particle sector

Let $\hat{P}$ be the projector onto the zero-phonon Hilbert subspace, which is spanned by the states $c_i^\dagger|0\rangle$, $\forall i$. The effective Hamiltonian in this subspace is, to second order in perturbation theory:

$$\hat{h}_1 = \hat{T} + \hat{P}\hat{V}\frac{1}{E_0 - \mathcal{H}_0}\hat{V}\hat{P},$$

where $\hat{T} = -t\sum_i(c_i^\dagger c_{i+1} + h.c.)$ is the bare kinetic energy, $\hat{V}$ is the bare particle - phonon coupling from Eq. (1) in the main text, and $\mathcal{H}_0 = \mathcal{H}_{\rm ph}$. The projection is straightforward to carry out and leads to:

$$\hat{h}_1 = \hat{T} + \hat{T}_2 - \frac{4g^2}{\Omega}\sum_i \hat{n}_i, \tag{5}$$

where $\hat{T}_2 = +t_2\sum_i(c_i^\dagger c_{i+2} + h.c.)$ is a phonon-mediated next-nearest-neighbor (NNN) hopping with $t_2 = g^2/\Omega = \lambda t/2$ (note the unusual sign). For convenience we define $\epsilon_0 = \frac{4g^2}{\Omega}$.

Setting $a = 1$, the polaron dispersion is, therefore,

$$E_P(k) = -\epsilon_0 - 2t\cos(k) + 2t_2\cos(2k).$$

It is straightforward to verify that if $t > 4t_2$, *i.e.* if $\lambda < \frac{1}{2}$, the polaron ground state (GS) momentum is 0. For $\lambda > \frac{1}{2}$, the polaron GS momentum is $k_P = \arccos\frac{t}{4t_2}$, going asymptotically to $\frac{\pi}{2}$ as $\lambda \to \infty$.

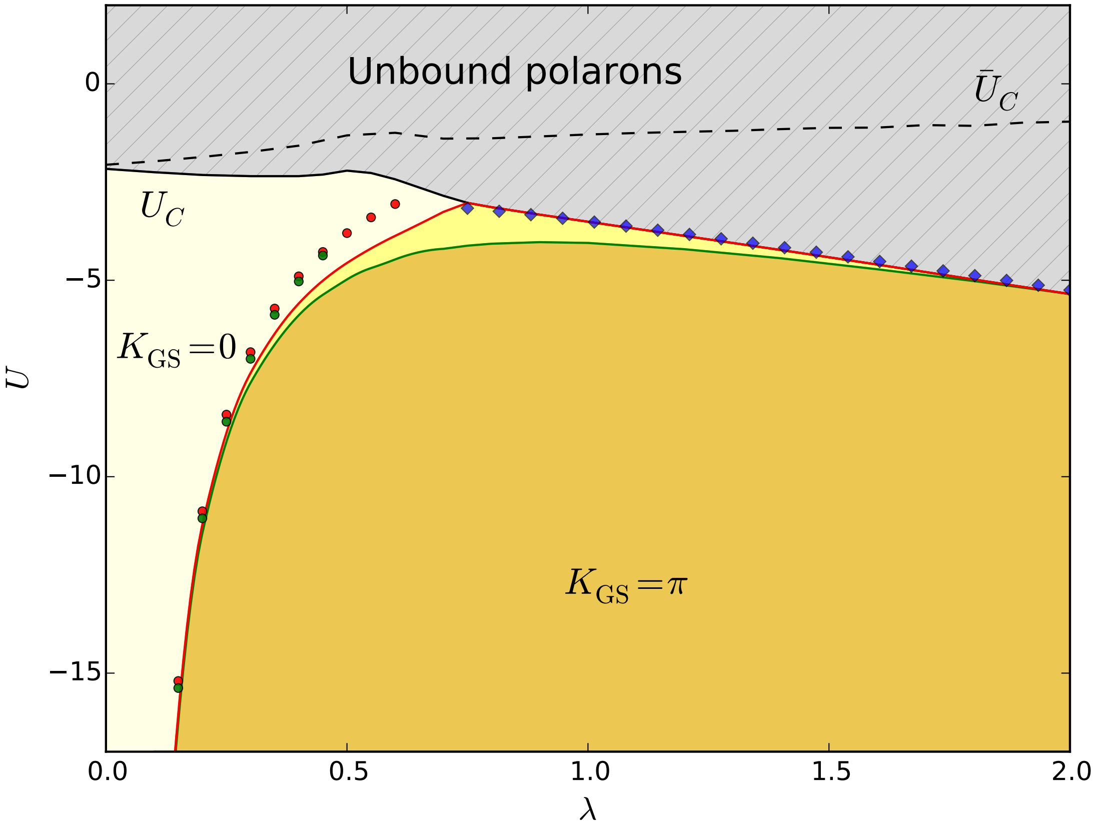

FIG. 1. (color online) Two-polaron phase diagram for $t = 1$, $\Omega = 100$. The solid black line shows $U_C(\lambda)$ below which stable bound states form, while the dashed line shows $\bar{U}_C(\lambda)$ defined in the main text; the difference between the two is the strength of the phonon-mediated repulsion. The red and green lines mark the sharp transitions of the bound dimer's GS. The lines are the VED results and the symbols of the corresponding color are the MA results. The diamond symbols are the PT results of Eq. (7). The area between the red and green curves corresponds to a dimer bound state with $0 < K_{\rm GS} < \pi$.

Two-particle sector

Repeating the projection onto the two-bare particle – zero-phonon subspace spanned by the states $c_i^\dagger c_{i+n}^\dagger|0\rangle$, $\forall n \geq 1, i$, we find:

$$\hat{h}_2 = \hat{T} + \hat{T}_2 - \epsilon_0 \sum_i \hat{n}_i + \tilde{U} \sum_i \hat{n}_i \hat{n}_{i+1}, \tag{6}$$

where $\tilde{U} = U + \epsilon_0$; U is the bare nearest-neighbor (NN) interaction.

The two-polaron bound state dispersion can be calculated numerically, either using direct diagonalization for a large-enough chain, or using the Equation-of-Motion (EOM) approach. We briefly present the latter and then use it to obtain analytical solutions in some specific limits.

We define $|K,n\rangle = \sum_i \frac{e^{iK(R_i+\frac{n}{2})}}{\sqrt{N}} c_i^\dagger c_{i+n}^\dagger |0\rangle$, $\forall n \geq 1$, and the propagators $g(n) \equiv \langle K,1|\hat{G}(\omega)|K,n\rangle$, where $\hat{G}(\omega) = (\omega + i\eta - \hat{h}_2)^{-1}$ is the resolvent of interest. The bound state energy (once a bound state appears) is at the lowest discrete pole of these propagators. Using the identity $\hat{G}(\omega)(\omega + i\eta - \hat{h}_2)^{-1} = 1$, we generate the EOM:

$$(\omega + i\eta - \tilde{U} + 2\epsilon_0 - \beta_K)g(1) = 1 - \alpha_K g(2) + \beta_K g(3)$$
$$(\omega + i\eta + 2\epsilon_0)g(2) = -\alpha_K[g(1) + g(3)] + \beta_K g(4)$$

and for any $n \geq 3$,

$$(\omega + i\eta + 2\epsilon_0)g(n) = -\alpha_K[g(n-1) + g(n+1)] + \beta_K[g(n-2) + g(n+2)].$$

Here, $\alpha_K = 2t\cos(\frac{K}{2})$, $\beta_K = 2t_2\cos(K)$.

The physically acceptable analytical solution for recurrence relations of this type is available in [9], however it is rather complicated and its poles cannot be extracted analytically. A general solution can be found numerically.

There are two cases that can be solved rather easily analytically, namely (i) if $\beta_K = 0$, and (ii) if $\alpha_K = 0$. The first is realized when $t_2 = 0$, and can be used as an indication of physics at very weak couplings $\lambda \to 0$. In this case the recurrence relation becomes trivial. For any $n \geq 2$ we have $g(n) = z(K,\omega)g(n-1)$ where

$$z(K,\omega) = \frac{1}{2\alpha_K}\left[-\tilde{\omega} + \sqrt{\tilde{\omega} + 2\alpha_K}\sqrt{\tilde{\omega} - 2\alpha_K}\right]$$

and $\tilde{\omega} \equiv (\omega + i\eta + 2\epsilon_0)$. This results in:

$$g(K,n,\omega) = 2\frac{[z(K,\omega)]^{n-1}}{\tilde{\omega} - 2\tilde{U} + \sqrt{\tilde{\omega} + 2\alpha_K}\sqrt{\tilde{\omega} - 2\alpha_K}}$$

for which exists a line cut that indicates a continuum (the two-particle continuum) for $|\tilde{\omega}| \leq 2\alpha_K$. A bound dimer appears if and only if there is a discrete pole at $\omega = E_D(K)$ such that a) $E_D(K) + 2\epsilon_0 < -2\alpha_K$ (the pole is below the continuum) and b) $E_D(K) + 2\epsilon_0 -$

$2\tilde{U} + \sqrt{E_D(K) + 2\epsilon_0 + 2\alpha_K}\sqrt{E_D(K) + 2\epsilon_0 - 2\alpha_K} = 0$ (the pole condition). It follows that the solution is

$$E_D(K) = -2\epsilon_0 + \tilde{U} + \frac{\alpha_K^2}{\tilde{U}},$$

which is below the continuum if and only if $\tilde{U} \leq -\alpha_K$. In particular, this requires $\tilde{U} < -2t$ for a bound state to emerge in the entire Brillouin zone, even at $K = 0$. This finite bare NN attraction is necessary in order to compensate for the lost kinetic energy, when the two particles bind.

The second accessible analytical solution is for $\alpha_K = 0$, which is valid for $K = \pi$ irrespective of the value of t, and therefore will give the exact PT bound dimer solution at the edge of the Brillouin zone. The solution follows as before, and it results in:

$$g(K, 1, \omega) = \frac{2}{\tilde{\omega} - 2\tilde{U} + 4t_2 + \sqrt{\tilde{\omega} - 4t_2}\sqrt{\tilde{\omega} + 4t_2}}$$

for which, repeating the analysis, we find that a discrete bound state appears if and only if $\tilde{U} < 0 \rightarrow U < -2\lambda t$, and its energy is $E_D(\pi) = -2\epsilon_0 + \tilde{U} - 2t_2 + \frac{4t_2^2}{\tilde{U} - 2t_2}$.

Note that the definition we employ for a *stable* bound state is not one that lies below the continuum at its momentum, but one that lies below the edge (lowest overall possible value) of the continuum. If this more stringent condition is not met, coupling to other fields (acoustic phonons, photons, ...) would allow transitions from this discrete state into the bottom of the continuum, so the dimer state is not truly stable.

To calculate U_C according to this definition, we compute the lower edge of the continuum. Given the single polaron dispersion $E_P(k)$ (calculated in the previous section), and noting that at a given total momentum K the two-polaron continuum start at $\min_q [E_P(K - q) + E_P(q)]$, one can easily solve for the lower edge of the continuum, E_c. One finds the result $E_c = 2 \min_k E_P(k)$, *i.e.* twice the polaron GS energy.

Requiring that $E_D(\pi) < E_c$ leads to

$$U_C(\lambda) = -2\lambda t - t - \frac{t}{2\lambda} + \mathcal{O}\left(\frac{1}{\lambda^2}\right). \quad (7)$$

This should agree with numerical results for sufficiently large λ where the GS momentum of the bound state approaches $K_{GS} = \pi$. However, note that λ should still be small enough such that PT remains valid. For example, for $\Omega = 100, t = 1$ this would require $\lambda < 2 \rightarrow g < 10$ such that $g/\Omega \ll 1$. For stronger couplings one needs to go to higher order(s) in perturbation theory.

Indeed, Fig. 1 shows good agreement at moderate and large $\lambda \leq 2$ between $U_C(\lambda)$ and this analytical prediction of Eq. (7).

---

[1] Berciu, M. Green's function of a dressed particle. *Phys. Rev. Lett.* **97**, 036402 (2006).

[2] Berciu, M. & Goodvin, G.L. Systematic improvement of the momentum average approximation for the Green's function of a Holstein polaron. *Phys. Rev. B* **76**, 165109 (2007).

[3] Goodvin, G.L. & Berciu, M. Momentum average approximation for models with electron-phonon coupling dependent on the phonon momentum. *Phys. Rev. B* **78**, 235120 (2008).

[4] Marchand, D.J.J. et al. Sharp transition for single polarons in the one-dimensional Su-Schrieffer-Heeger model. *Phys. Rev. Lett.* **105**, 266605 (2010).

[5] Adolphs, C.P.J. & Berciu, M. Strongly bound yet light bipolarons for double-well electron-phonon coupling. *Phys. Rev. B* **90**, 085149 (2014).

[6] Berciu, M. & Fehske, H. Momentum average approximation for models with boson-modulated hopping: Role of closed loops in the dynamical generation of a finite quasiparticle mass. *Phys. Rev. B* **82**, 085116 (2010).

[7] Sous, J. et al., in preparation.

[8] Berciu, M. Few-particle Green's functions for strongly correlated systems on infinite lattices. *Phys. Rev. Lett.* **107**, 246403 (2011).

[9] Möller, M., Mukherjee, A., Adolphs, C.P.J., Marchand, D.J.J. & Berciu, M. Efficient computation of lattice Green functions for models with longer range hopping. *J. Phys. A: Math. Theor.* **45**, 115206 (2012).
